# Implementation Process and Impacts of a Participatory HIV Research Project with Key Populations

**DOI:** 10.1155/2018/5845218

**Published:** 2018-05-31

**Authors:** Sónia Dias, Ana Gama, Daniel Simões, Luís Mendão

**Affiliations:** ^1^Global Health and Tropical Medicine (GHTM), Instituto de Higiene e Medicina Tropical (IHMT), Universidade Nova de Lisboa (UNL), Portugal; ^2^Grupo de Ativistas em Tratamentos (GAT), Portugal

## Abstract

A participatory HIV research project was conducted with sex workers (SW) and men who have sex with men (MSM) to understand epidemiological HIV dynamics and associated sociobehavioural factors among these vulnerable groups. We examine the impact of this project, critically analysing the processes undertaken and focusing on the advantages and the challenges faced. A partnership was built through two Community Advisory Boards (CABs) and a Scientific Commission (SC). Regular meetings, workshops, and focus groups were conducted with CABs, SC, and partners to assess the processes and outcomes of the project implementation. This participatory research produced change processes with impacts at different levels: individuals, community organizations, health professionals, academics, and policy-makers. Advantages of the participatory process were encountered but also challenges, evidencing the dynamic and complex nature of each project's stage. This project showed that participatory research can work as an intervention. Indeed, it triggered a dynamic and interactive process of knowledge coproduction and translation into effective community-oriented health actions and policies. The participatory research reproduced an innovative alliance for HIV prevention and sexual health promotion responsive to local needs and priorities. Further efforts are needed to systematize and evaluate the processes and impacts of participatory health research.

## 1. Introduction

The production of evidence that informs effective and sustainable health policies and actions is key to improving populations health and obtain health gain [1]. Several authors advocate that such evidence produced through health research should (1) have quality, be focused on the health problem but also be based on its context and the involved actors; (2) be relevant, i.e., meaningful for affected communities as well as beneficial, constructive, and useful; and (3) have applicability, be usable or implementable in order to improve health practices, health outcomes, and conditions affecting health [1, 2]. This is particularly relevant in the context of most vulnerable populations, who are frequently exposed to conditions and spaces of vulnerability that reinforce their vulnerability process and distinguish them from the other populations, which translates into health inequities [3].

This is the case of sex workers (SW) and men who have sex with men (MSM) who are considered most vulnerable in the field of sexual health, namely, sexually transmitted infections as HIV infection [4–6]. Over the last decades, despite HIV infection having evolved into a chronic disease with effective combination of antiretroviral therapy [7–9], HIV infection continues to be a concern in global public health. In the European Region, Portugal had the second highest HIV prevalence—0.7%  [0.6–1.0%] (among adults aged 15–49 years old)—according to the most recent available estimates [10]. In low-level and concentrated epidemics as in Portugal, SW and MSM are considered key populations who, due to specific higher-risk behaviours in combination with interpersonal, sociopolitical, and cultural contexts, are at increased risk of HIV [11, 12]. These most vulnerable populations are often subject to social stigma and marginalization which renders them particularly hard to reach [4–6]. Indeed, health research has struggled with the inability to reach and cover such “hidden” populations who, despite being vulnerable to poor health, are frequently understudied and missed in conventional surveillance efforts [3–6]. Given the frequent inability to reach and recruit participants from these most vulnerable subgroups and obtain their perspective about the problem/phenomenon under study, the gap between knowledge producers and studied populations is often complex to overcome. The challenges in reaching and covering most vulnerable populations in research have called for a different paradigm of doing research, giving special attention to the participatory approach in health research.

In this context, based on a joint partnership between an academic institution and an HIV/AIDS nongovernmental organization with years of community work experience, a participatory HIV research project called PREVIH-*HIV/AIDS infection in Sex Workers and Men who have Sex with Men: Prevalence, determinants, prevention interventions and access to health* was developed in Portugal. The general aim of the project was to produce knowledge that contributed to the promotion of sexual health, to reduce the transmission of HIV infection and to improve access to healthcare among SW and MSM. Simultaneously, it intended to promote capacity building of stakeholders for advocacy and policy-making and to empower communities to develop skills for sexual health promotion. With an intersectoral nature, this project involved all the stakeholders interested in and affected by the HIV problematic: research/education institutions, policy-making institutions, health services, nongovernmental organizations (NGOs), community-based organizations (CBOs), and civil society. In a global perspective, the evidence produced informed the design and implementation of HIV prevention interventions in a subsequent component of the project.

In literature, participatory research is defined as a collaborative approach that involves equitably community members, representatives of governmental and nongovernmental organizations, and researchers in the process of knowledge production, incorporating the different perspectives and experiences of these stakeholders [13, 14]. Each partner contributes with unique resources and shared responsibilities to the understanding of the phenomenon being studied and its sociocultural dynamic [13, 14]. This approach combines research with capacity building strategies in order to reduce the gap between the produced knowledge through research and the translation of that knowledge into interventions and policies that improve populations health [13, 15].

Conducting research in collaboration with the communities increases the study population's adhesion to the studies, which allows reaching the most vulnerable populations and obtaining spontaneous and reliable information from participants. This advantage is even more relevant given the fact that HIV and sexual risk behaviours, being strongly shaped by culturally based factors as social norms and values related to gender and taboos, are particularly sensitive topics for some communities [16, 17]. Additionally, using the participatory approach in sexual health research with most vulnerable populations has the potential for producing greater knowledge, incorporated with the perspective of communities and translatable into effective sexual health promotion policies and strategies, more adapted to the communities' needs [13, 18–21]. Indeed, the collaborative nature of participatory research, in which communities act as active partners in the identification of the key problems, in the implementation of the methods and in the utilization of research results, has contributed to the collection of relevant and useful information from communities for improving their health [22].

In a dynamic, ecological-systems perspective, the participatory approach in health research is valuable to address the complexity, multifactoriality, and multidimensionality of health problems, framed in population-setting systems [23]. This draws attention, not only to the system context, but also to the linkages and interactions among the system's parts, especially the networks of social relationships that make up the system, the variety of roles that exist or can be created within those networks, the status conferred on those roles, the symbolism, and the meaning that different actors draw from the intervention event [23]. In this sense, alliances and coalitions that are inclusive of a diversity of actors are essential features of public health research and intervention [24].

Health research with a participatory approach can be considered as a time limited new activity setting with the potential to produce changes in the system [23]. In this sense, participatory approach can impact evolving networks of person-time-place interaction, changing relationships, displacing existing activities, creating new roles and redistributing, and transforming resources across the network [23]. The result of this collaborative and action-oriented process is the enhancement of capacity building and empowerment of communities to address and meet their health priorities [25].

Despite the increasing attention to this approach, the changes that occur when participatory research is placed into action are not fully understood. Additionally, comprehending what happens during participatory research implementation contributes to effectively linking research events to outcomes [26].

In this article we examine the impact of a participatory HIV research project with SW and MSM. We critically analyse the processes undertaken during its implementation, focusing on the advantages, the challenges encountered, and the extent of the results of this participatory research.

## 2. Methods

In order to ensure a strong involvement of key stakeholders in the project and considering the diversity of the study groups, at the start both initial promoters conducted an exhaustive mapping of NGOs and CBOs that worked with SW and MSM nationwide in an attempt to embrace a high and diverse number of potential partners to collaborate in the project. Each organization filled in a questionnaire providing information on the actions they undertook and the areas being reached. Organizations also indicated focal points of linkage with PREVIH team. Two Community Advisory Boards (CABs) were formed comprising social intervention workers, NGOs representatives, and members of the communities: one CAB with the Civil Society and SW Community and one CAB with the Civil Society and MSM Community. The SW CAB initially involved social intervention workers who worked in the field with SW and had expertise and knowledge on strategies of intervention. Starting from the outreach structures, the project team described the principles, objectives, and importance of SW to participate in the CAB and asked these structures to invite their users to integrate it. This way a group of elements from the SW community was integrated into the SW CAB, which comprised 7 SW and several organizational representatives. The MSM CAB comprised representatives of organizations and LGBT associations, community leaders, and 14 MSM. For validation and monitoring of the project, a Scientific Commission (SC) was constituted with several specialists and experts with long experience in research and intervention, such as academics in the areas of epidemiology, public health, and social sciences, professionals from diverse health institutions and NGOs working with SW and MSM populations, and representatives of policy-making institutions.

The formation of both these structures (the CABs and the SC) was the base for building a partnership with all stakeholders. After establishing the partnership, CABs meetings were held for deep discussions between partners on the overall purpose and strategies of the project, in order to achieve meaningful consensus of the project's focus and boundaries.

At the start, formative research was carried out in close collaboration with partners, focusing on SW and MSM communities, to assess the feasibility and acceptability of research activities, the accessibility of health services, and prevention needs. This included a mapping of MSM socialization venues/sex work venues and networks; an assessment of implemented intervention activities and coverage; an assessment of information, education, and communication (IEC) materials followed by a revision of its content; an assessment of information lacking and strategies to reach those who need information; a mapping of the HIV services/organizations working with SW and MSM; and an assessment of accessibility, services provided, and social attitudes towards SW/MSM.

The participatory research was conducted through a biobehavioural survey to assess HIV prevalence, understand its social and behavioural correlates, and characterize the access to HIV health services. All partners—governmental and nongovernmental organizations, community members, and health services—participated in defining the objectives of the studies, outlining the methodological procedures, designing the questionnaires, implementing the field work, and interpreting the results. Methodological procedures are described elsewhere [27, 28]. In brief, a snowball sampling method was used in an attempt to reach hidden subgroups of SW and MSM. Data were collected by interviewers (members of NGOs, CBOs, and SW/MSM civil society) who participated in interviewer training sessions and were coached in collecting quality data. The data collection instrument consisted of a closed-ended questionnaire with items on sociodemographics, sexual health, risk behaviours, and access to health services. After completing the questionnaire, an HIV rapid test was provided to respondents. Facing the possibility of dealing with HIV-positive cases, a referral system for appropriate healthcare was ensured based on an effective cooperation with the health services. Finally, all participants and SW/MSM approached were given a prevention kit containing condoms, lube, and informative leaflets on HIV prevention and testing. This participatory research was approved by the Ethics Committee for Health of the North Regional Health Administration. Regular meetings with the CABs and the SC were conducted throughout the project in order to discuss and establish the appropriate methodology for the SW and MSM populations and settings ensuring the quality and rigor of the project.

The findings obtained provided useful information and tools to develop a subsequent component of HIV prevention interventions. Therefore, after the survey, two workshops with key actors (some of them were members of the CABs and the SC) were conducted to ultimately debate upon the questions “How to understand and use the results in the communities' own settings?” and “How to put the results into practice and translate them into effective interventions?”

The following component of PREVIH involved the design and implementation of innovative evidence-based interventions for sexual health promotion, HIV prevention, and promotion of access to healthcare. These interventions covered HIV services/organizations mapping, IEC activities, peer education, and HIV testing initiatives in community-based settings.

A critical analysis of all processes was produced through focus groups with key informants who were members of the partnership and represented diverse stakeholders. Focus groups were chosen as the most appropriate method to explore the impact of new practices on roles, activities, processes, and perceptions and examine interactions among participants [29, 30]. The organization of the focus groups was decided in collaboration with the community partner. The six group discussions occurred throughout the project in key moments before, during, and after the project's implementation. In each phase, two focus groups were conducted with 8–10 participants each: one group with community members and representatives of community organizations and one group with policy-makers, health professionals, and academics. The organization of focus groups based on relative homogenous backgrounds was key to facilitating communication within the group. The group discussions focused on the assessment of the processes of the project implementation, the innovative practices taking place within the network, and the advantages and challenges encountered throughout the project.

## 3. Results

Throughout time, the development of this participatory project produced change processes with impacts at different levels. Along the way, clear advantages were found but also several challenges were encountered, evidencing the dynamic and complex nature of each stage of this HIV project.

### 3.1. Establishing the Partnership

From the start, an effort was made to embrace a high and diverse number of partners to collaborate on the project, ensuring that the principles of active participation, ownership, and empowerment were transversal and constant throughout the project. However, this initial process occurred differentially with the SW group and the MSM group. On the one hand, the context of organized MSM social movements and community organizations made an easy task for the PREVIH team to identify community partners and later to conduct the formative research. In contrast, the SW civil society was not organized nor was easily recognizable, and therefore greater efforts were demanded to reach and involve this community in the project. From the beginning, the NGO promoter of the project acted as mediator between the project and the SW community until the “trust point” was achieved. This articulation was less complex with the MSM community. Additionally, based on the professional links with policy-makers and health professionals, the initial promoters of the project invited representatives of these stakeholders to the partnership. Building this comprehensive partnership helped create a setting conducive to making new links, connections, and exchanges between partners, especially reducing the gap between policy-makers and communities.

Overall, this first phase was essential to render the context more favourable and open to the project. At the end, the project partnership embraced diverse and numerous partners: 22 NGOs/CBOs spread nationwide, the regional health administrations of the entire country (governmental institutions), 10 public early HIV detection centres, 6 hospitals and other public health structures (specialized HIV/STI care services), over 20 different experts from key scientific fields (Epidemiology, Public Health and Social Sciences), and over 50 MSM and SW community members.

From the start of the project some challenges were foreseen, namely, the difficulty of starting research with little knowledge on such understudied populations and the fact that relevant NGOs were poorly organized (often fragmented or isolated) and had implemented sporadic actions oriented to SW and MSM. Pertinent questions arising during this stage and requiring critical analysis included the following: which organizations best represented the communities and which ones should be included in the partnership? How would the previous relationship between the different organizations have an influence? How to deal with noninterested/opponent organizations? The level of community participation varied from situations in which partners were actively involved in most stages of the research to ones in which, according to their will, partners were only consulted and kept informed about issues of interest to the research. Most partners lacked experience and preparedness to work in participatory research projects and some partners expected to have a more passive participation as top-down models are common. This demanded partners' adaptation to a new system of collaborative working, more time, and dialogue to increase participation. These background circumstances paved the way to establishing from the starting point a synergistic partnership between academic and community partners to design the project and take on shared governance.

Also, having numerous stakeholders to consider delayed some of the project's components. Some partner organizations were understaffed, which required an effort from them to stay involved. Overall, to maintain an active partnership (with different competencies, degrees of commitment, interests, motivations, expectations of the project outcomes, and the need of continued adjustments to the project) required time, dialogue, resources, and increased workload.

### 3.2. Conducting the Participatory HIV Research

In the first meetings in partnership, the debates on the research overall purposes and strategies allowed all partners to rethink and redefine the substantive issues underlying the project and address specific SW and MSM concerns. This enhanced the relevance of the main health questions of the project to the community and communities' awareness of the importance of knowledge production to improve health. Yet, achieving a full commitment of community partners on the project's relevance and ensuring that they acknowledged the project as a priority were challenging. The integration of stakeholders as partners led them to act as negotiators between the project's objectives and their own objectives. Multiple preconceptions/prejudice from communities, NGOs/CBOs, academics, health professionals/services, and policy-makers/state had to be addressed. It demanded a continuing effort to build and maintain mutual trust and respect and to achieve agreement between academic scientific methods and civil society commitment with advocacy for SW and MSM rights. One example of this effort was an initial discussion within the MSM CAB on the term used to designate the study population—MSM. Though in the academic context “MSM” has been broadly adopted in research, for the community this was considered a reductive expression to characterize a heterogeneous group based on the single feature of sexual partners' sex. These discussions allowed reducing defensive stances from partners, addressing myths and misconceptions, and reaching a consensual definition of the target population that could be operationalized to respond to the research questions.

Globally, the discussions in partnership about the procedures and implementation of the research led to developing a more appropriate study design, methodology, methods, and measures for the SW and MSM populations and settings. More specifically, through CABs auscultation, all partners participated in the definition and revision of the items to be included in the questionnaire. During this process misinterpretation of several questions was detected. For example, while discussing the instrument within the SW CAB disparate definitions of “occasional” and “regular” sexual partner emerged. Debating these issues with partners was valuable to bridge differences, ending up having contextually congruent concepts and measures. Ultimately, this process helped adapt the instrument to the study populations' characteristics and context.

The meetings with both CABs enabled consulting members about obstacles and strategies to best reach and involve SW and MSM hidden subgroups in the survey assuring scientific rigor. For example, in a pilot phase a high rate of noncompliance with the study was observed among SW and MSM and the discussions with the CABs allowed bringing up some strategies to alter this trend. An agreed upon strategy was to include elements of the study populations (MSM and ex-SW) in the interviewers' team, and this helped enhance the acceptance of the study and reach hidden subgroups that were out of range of the existing structures.

In the field, the implementation of the snowball sampling method was possible through the social networks of community partners and their expertise in the field. The interviewer training sessions for data collection were dynamic, with feedback being collected from all participants and taken into account. This increased capacity of trainees as they actively participated in the debate about, for example, dubious questions/answers and terminology used in the questionnaire, the approach to invite potential participants, and procedures to obtain the informed consent and to administer the questionnaire.

Overall, the collaboration of community partners in advertising the project, recruiting participants, and collecting data as interviewers enhanced the communities' acceptance of the study, the recruitment and retention rates of participants, and the diversity of participants by including hard-to-reach subgroups. The meetings with the SC conducted throughout the project to discuss and validate the methodological options and the monitoring and evaluation of its implementation ensured the quality and scientific rigor of the project, as well as its relevance and responsiveness. At the end of this phase, 60 SW peers/representatives of NGOs and CBOs working with SW and 31 MSM (peers and NGO/CBO members) had been enrolled as interviewers. Total samples of 1040 SW and 1046 MSM respondents were reached, over 1100 HIV rapid tests were performed, and about 3400 people received HIV information and prevention material.

This kind of research that focuses on sensitive topics like HIV among most vulnerable populations raises relevant ethical issues related to human rights, social inequities, potential harm and impact in terms of social stigma/discrimination, and protection of participants' anonymity and data confidentiality. In this context, using a participatory approach also allowed addressing and discussing these crucial aspects within partnership.

### 3.3. Analysing, Interpreting, and Disseminating the Results

After collecting the data, the presentation of the preliminary results to the CABs and the SC stimulated the discussion on the relevant analyses and the interpretation of the findings. Although the members of the partnership had different backgrounds and competencies, acknowledging and respecting each other's contribution allowed creating new synergies and discovering possibilities for knowledge and action. Members outside the academic institutions had a meaningful role, providing important insights into additional analyses to further explore the complex links between variables that were not initially foreseen. Indeed, discussing the findings within the partnership allowed the integration of multiple perspectives that enriched the interpretation and contextualization of the results and enhanced a process of exchange between community stakeholders and researchers. The dissemination of the results was another aspect discussed in partnership. Controversy arose during this period, especially with an ethical “dilemma” in the centre of the discussion: while the evidence on HIV prevalence and associated factors among SW and MSM would be crucial to inform health policies and prevention strategies, the risk of consequent social prejudice and stigma towards SW and MSM populations was real and this required serious debate within the partnership. Discussing the dissemination strategy with partners enabled the presentation of the results to diverse and wide audiences beyond the scientific community (policy-makers, health professionals, SW/MSM communities, and civil society). Some examples included a public conference coorganized by academic and community partners with visibility in social media, as well as “Community Reports” presented and distributed by the community partners. In addition, the main findings were presented in scientific conferences and were published in peer-review scientific journals where community partners were included as co-authors [27, 28]. In addition, the project constituted a data source for response to specific health and HIV indicators by international institutions like UNAIDS, ECDC, and WHO and national institutions like the General Directorate of Health and the National Institute of Statistics.

### 3.4. Designing and Implementing HIV Prevention Interventions

The interventions developed in a subsequent component of the project attempted to adequately respond to communities' specific needs identified through the participatory research. The planning and implementation of the different interventions in close partnership with the NGOs, CBOs, and civil society partners enabled promotion of institutional capacity building on advocacy for sexual health promotion and rights. It also enhanced the empowerment and the capacity of SW/MSM communities to understand their health needs and redirect their efforts in addressing them. The developed interventions comprised HIV services/organizations mapping, IEC activities, peer education, and HIV testing initiatives in community-based settings.

The systematic mapping of HIV services and organizations nationwide working in the HIV field, particularly with SW and MSM, provided a knowledge base that contributed to promoting the creation of collaborative partnerships for the development of multisectoral interventions as effective responses to complex problems such as HIV, in the future.

The elaboration of IEC materials was based on the partners' contribution to the revision and assessment of existing IEC materials, which helped improve the appropriateness and effectiveness of IEC materials. This intervention also covered the promotion of campaigns for HIV information and prevention through the distribution of IEC and prevention materials within SW and MSM communities, using innovative and creative methodologies. The IEC materials were made available to NGOs/CBOs and institutions working with SW and MSM. This approach helped prevent duplication of efforts across the NGOs and CBOs that implement IEC activities in their daily activities and allowed developping more targeted and effective initiatives by providing the information that is really needed.

The peer education initiatives contributed to promote changes in knowledge, skills and competencies, reduction of risk behaviours, and access to healthcare. Those initiatives were implemented by members of the SW and MSM peer group who were influential in eliciting behavioural change among their peers. A total of 25 MSM peer educators trained other peers to disseminate risk reduction information within their social networks. Among SW, six peer educators were trained and were integrated in a semiprofessionalized fashion on NGOs outreach teams in which individual goals and evaluation plans were designed collaboratively. Moreover, based on the connections built between these SW, a network of SW was established with the aim of recognition and valorisation of sex work in the country.

Also, new HIV testing strategies were collaboratively designed and developed by community partners aimed to improve access to HIV health services to subgroups that often did not access formal healthcare due to stigma and discrimination-related barriers. HIV testing was traditionally only available at formal healthcare services, but the knowledge produced from this project raised stakeholders' awareness, especially policy-makers, towards the urgency of developing HIV testing strategies that would cover those most vulnerable and out of reach of existing health structures. Examples of such initiatives were the establishment of new proximity responses such as mobile units and community-based centres for HIV testing and counselling targeting those most vulnerable (including sex workers and injection-drug users).

In this interventional phase, adjustments to activities were required according to each area and population and often to organization/community partner. Several “subprojects” with specific implementation procedures and processes were created, which increased the project workload. Overall, the involvement and participation of community partners in designing and implementing the interventions favoured the creation of a context to promote sexual health and advocate for changes in public policies that have an adverse impact in their communities.

## 4. Discussion

The experience of implementing this HIV research project within a participatory approach reinforced its potential for producing relevant knowledge that contributes to effectively improving populations' health, as documented elsewhere [31]. Through the active participation of key stakeholders in this HIV research project it was possible to reach and gather large and heterogeneous samples of SW and MSM, including hard-to-reach subgroups at increased risk of HIV infection, in a context where many of these populations have a feeling of mistrust and disinterest towards research. Indeed, the obtained sample of 1040 SW was comprised of outdoor and indoor SW and nationals and nonnationals, with poor socioeconomic status and reported HIV/other STIs. This research also reached a subsample of transgender SW, a subgroup who is frequently missed in research initiatives and is particularly at increased risk of HIV infection [32]. Moreover, the 1046 MSM enrolled in this research included men with diverse sexual identities and orientations, a feature that has been described as an individual-level factor associated with HIV risk behaviours. Overall, through this HIV research project it was possible, for the first time, to obtain information on the HIV prevalence and associated factors among key populations in the country that were not being covered in traditional surveillance efforts and therefore reliable information had not existed thus far.

The integration of the perspectives and experiences of communities enabled deeper understanding of the complex processes underlying the multiple interdependent sociocultural and contextual factors associated with HIV infection. For instance, it allowed better comprehension at what extent contextual factors, such as poverty, drug use, and sex work settings, are linked and increase SW vulnerability to HIV infection and what contexts increase MSM exposure to HIV risk, especially considering cruising venues and other locations where MSM seek sexual partners [27, 28]. Ultimately, this process of collaborative knowledge production enabled generation of more relevant knowledge translatable into practices that effectively responded to these populations' needs. In this sense, the recommendations driven from this project were contextually and culturally grounded and relevant, enhancing the applicability of the research findings into more effective community-oriented health actions and policies. Taking shared decisions contributed to strengthening the partners' sense of ownership, regulation, and coresponsibility, while enhancing trust, legitimization, and credibility of the project by nonacademic partners.

This participatory research worked in and of itself as an intervention as it increased all actors' capacity to deal with the HIV issue. At individual and civil society level, there are now more people aware and committed to HIV issues, with access to HIV prevention information, tested for HIV and aware of their serostatus, and informed about where to get tested and treated. Community-based organizations have developed a more reflexive practice, increased capacity for monitoring their activities, and are better informed to address communities' health needs and advocate for sexual health rights. Health professionals are more aware of further steps to improve the adequateness of healthcare delivery. Academics are now more knowledgeable of the epidemiological HIV dynamics; the best strategies do reach most vulnerable populations and are better prepared to produce evidence to inform sexual health rights-based policies and HIV prevention interventions. Researchers with different backgrounds worked together to synthetize existing evidence on this theme. By drawing on evidence across different contexts, disciplines, and actors, academics could make stronger arguments for policy change, along with increasing their expertise and being seen as credible by policy-makers. In their turn, policy-makers have better understanding on the contexts of vulnerability, what policies and strategies work, and what does not work and why.

Nevertheless, several challenges in adopting the participatory approach in this HIV research were experienced and should be considered in the future. Sharing power and ensuring an equitable involvement of the different partners in decision-making were lengthy and demanding processes [33]. Maintaining an effective partnership, which is a fluid, evolving process, required continuing effort and negotiation skills. These challenges were experienced by the research team. Academics are generally used to having the control of the research process. To share power and negotiate with partners and be available to make adjustments along the way in this participatory project implied flexibility. In fact, the focus groups conducted to assess the project's implementation served also as a tool and an opportunity for the research team to exercise their self-reflexivity on the effects of the participatory processes undertaken and the adaptations needed throughout the project. Another common challenge was related to the different partners' priorities. For example, the academics were committed to more traditional methods that ensured the scientific rigor of research, were pressured by the need to quickly publish the evidence in high-impact scientific journals, and prioritized research topics that they considered more interesting and appealing to investigate and publish. On the other hand, some community partners were resistant to research that challenged their values, attitudes, and practices, were not sensitized to the methodological procedures required, and were committed to respond to their specific real concerns. Divergence and controversy arose while achieving a compromise to meaningful consensus that implied negotiation between conflicting interests. Globally, the process forced actors to reorient and expand the problematization, considering their multiple perspectives of analysis of the project and different interpretations of its successes/failures.

PREVIH operated in a singular context of different actors (academics, community representatives, NGOs/CBOs, health professionals, and policy-makers) reconfigured in terms of their identities, interests, and practices, confirming that participatory research has the potential to intervene and transform the system by its interaction with the context and the capability created from that interaction [23, 26]. Through a reflexive dynamic, this participatory research reproduced an innovative alliance for HIV prevention and sexual health promotion responsive to local needs and priorities [24]. The creation of new connections between partners changed social positions, some people becoming more central, others less central, and others connected for the first time. These connections created new opportunities for the exchange of information, material resources, and support. In other words, the created network facilitated the exchange of diverse forms of knowledge and knowing and enabled the development of shared understanding and insight between initially foreign universes, which, in turn, gradually reconfigured themselves through their interactions, as described by other authors [23, 24].

This experience confirmed that rather than the conventional view of research as a “program package” standardised and replicable across settings, research should be understood as a dynamic process [23]. In fact, innovative practices took place throughout the project and were crucial to the coconstruction of knowledge among the actors at different levels, as theorized by other authors [29] as practices at cognitive level: circulating partners' knowledge which helped to frame research questions, knowledge production processes, and translation into the various partners' networks; strategic level: all activities, tools, and competences mobilised to raise and maintain the different partners' interest in participatory research; and logistic level: the coordination tasks that create the actual conditions for the partnership. Indeed, the discussions promoted within the CABs and the SC at key stages of the project were a major mechanism to enhance partners' capacity building. The interaction that was created between the different elements of the CABs generated mechanisms of ownership and empowerment among these groups, in which each other's experiences and knowledge were valuable resources for the partnership performance. Additionally, having CABs enabled the proximity to the study populations, increasing communities' adhesion and the appropriateness of activities, which ultimately helped ensure that the project would permanently respond to the communities' needs and concerns.

The emergence of this new structure for action aimed to jointly promote sexual health, reduce the transmission of HIV infection, and improve access to healthcare among SW and MSM, which represented a system-level impact of the participatory approach. Besides the individual units of change within the system, the knowledge and skill sets changed in the partners. The new structure potentially represents a particular new capability and its outcome is other vital new connections made both within the original network and outside of it, which continually place the actors in the network in a position to access resources and opportunities for adaptation and growth, as described elsewhere [23].

This experience also triggered a dynamic and interactive process of knowledge translation, i.e., of knowledge production and application into effective actions aimed to improve populations' health. The PREVIH experience led to identifying specific community needs and opportunities to intervene. In this sense, outcomes not foreseen initially emerged such as the development of new HIV testing policies, networks, and initiatives that continued after the project's timeline ended.

## 5. Conclusions

This experience enabled the construction of innovative alliances for coproduction of knowledge adapted to the needs of involved actors and translatable into effective sexual health promotion and HIV prevention interventions. This participatory health research enhanced partners' capacity for conceiving actions more relevant, coherent, responsive, and sustainable overtime.

This project reinforced the relevance of the participatory research as an alternative approach to address the current challenges in health research and tackle health inequities. Indeed, the complex nature of health problems, the diversity of settings, and the disparate levels of vulnerability across populations highlight the need of innovative ways to conduct research collaboratively, which calls all stakeholders to “step outside of the box.”

Though some challenges remain, how to move forward to better systematize and evaluate the processes and impacts of participatory health research in its different complex dimensions, measure the dynamic changes, underlying shared values and principles, and assess capacity building in network, among others, is still challenging. This implies that stakeholders are skilled to undertake new practices and funders are aware of the need for further resources allocation.

Overall, in the face of the innumerable strengths and potentialities of participatory health research, to scale up this approach while tackling its challenges is key to maximizing its impact in improving populations health and promoting health equity.
